# The Design and Implementation of a High-Precision Positioner Fixture

**DOI:** 10.3390/mi12101227

**Published:** 2021-10-08

**Authors:** Xuesen Zhao, Rongkai Tan, Zhe Wang, Xicong Zou, Zhenjiang Hu, Tao Sun

**Affiliations:** 1 Center for Precision Engineering, Harbin Institute of Technology, Harbin 150001, China; wangxiaozhe1@gmail.com (Z.W.); huzhenjiang@hit.edu.cn (Z.H.); taosun@hit.edu.cn (T.S.); 2 School of Mechatronics and Vehicle Engineering, East China Jiaotong University, Nanchang 330013, China; 3 Jiangxi Provincial Institute for Drug and Food Control, Nanchang 330029, China; 4 Hefei Institutes of Physical Science, Chinese Academy of Sciences, Hefei 230031, China; 5 School of Mechatronics Engineering, Heilongjiang University, Harbin 150080, China; zouxicong@hlju.edu.cn

**Keywords:** positioner fixture, high-precision, repeated positioning accuracy, high stiffness

## Abstract

In this paper, a novel positioner fixture with a high repeated positioning accuracy and a high stiffness is proposed and investigated. A high-precision end-toothed disc is used to achieve the high repeated positioning accuracy of the designed positioner fixture. The mathematical models of the cumulative error of the tooth pitch, the tooth alignment error and the error of the tooth profile half-angle of the end-toothed disc are analyzed. The allowable tolerance values of the cumulative error of the tooth pitch, the tooth alignment error and the error of the tooth profile half-angle of the end-toothed disc are given. According to the theoretical calculation results, a prototype positioner fixture is fabricated and its repeated positioning accuracy and stiffness are tested. The test results indicate that the stiffness of the proposed positioner fixture is 1050.5 N/μm, which is larger than the previous positioner fixtures of the same type. The repeated positioning accuracy of the proposed positioner fixture in the x, y and z directions are ±0.48 μm, ±0.45 μm and ±0.49 μm, respectively, which is significantly higher than the previous positioner fixtures.

## 1. Introduction

In the machining process, the fixture is the most used auxiliary tool and the accuracy and reliability of the fixture directly affect the processing efficiency and the manufacturing accuracy of the part [1,2,3]. Studies show that about 40% of parts that are rejected are mainly because of dimensioning errors due to poor fixture designs [4]. Furthermore, the parts need to be demounted and installed for exchanging between different machining procedures and a transposition error value is generated in this process. Therefore, the reduction of the positioning error caused by the clamping process is very important to the forming accuracy of the part. A high-precision positioner fixture can ensure that the positioning reference of the part is unchanged when the processing station is changed, thereby reducing the positioning error caused by the clamping process as much as possible [5,6]. In the field of precision machining, most parts are produced in small batches; this is particularly the case for customized parts, which need to be demounted and installed frequently during the machining process. If positioning errors caused by the disassembly and installation processes cannot be controlled, it is difficult to realize the final machining accuracy of the part [7,8]. Therefore, a high-precision positioner fixture is indispensable for precision machining.

The design process of conventional fixtures is usually divided into four sub-steps: (1) setup planning, (2) fixture planning, (3) unit design and (4) verification [9,10]. In addition, computer-aided technology has been widely used for improving the fixture design process since the 1980s [11,12,13,14]. The sub-step design process of fixtures and the application of computer-aided technology simplifies the fixture design, especially for fixture configuration design [1]. Zhou et al. proposed a feature-based fixture design methodology; previous fixture design cases and design rules are described in association with features and thus the design knowledge is integrated with the geometric information of aircraft structural parts, which improves the efficiency and quality of the fixture design [13]. Peng et al. proposed a new fixture design methodology where the rule-based reasoning and case-based reasoning methods are combined for a machining fixture design in a virtual reality-based integrated system [15]. However, the stiffness and repeated positioning accuracy of the fixtures are less considered in the computer-aided fixture design methodology. Scholars have conducted much research on positioner fixtures for meeting the different applications and needs in the manufacturing market. Kartik et al. proposed a mobile positioner fixture based on three high-precision steel balls to achieve the positioning [16]. The three steel balls are distributed in an equilateral triangle and it is matched with a V-shaped structure that can move in the groove so the adjustment of position and direction can be realized. The experimental results demonstrate that the positioner fixture had a good repeatability and high stiffness stability [17]. Yingguang et al. designed a responsive fixture based on product dynamic detection technology and the test results indicated that the deformation of the workpiece during processing was reduced by using the responsive flexible fixture [18]. However, a high machining accuracy was achieved through online monitoring and the adjustment of the fixture deformation. Thus, the fixture had a complicated structure and a high manufacturing cost. The research of Gonzalo et al. indicated that the main factors affecting the accuracy of parts are static deformation and dynamic vibration and static deformation is mainly the deformation caused by the clamping force and the cutting force [19]. Pellegrinelli et al. proposed a method to quickly replace the part substrate. Based on the flexibility provided by the positioner fixture, the substrate could be quickly switched between different machine tools [20]. It should be noted that the stiffness and repeated positioning accuracy of the positioner fixture were not considered in this paper. Several companies have also developed commercial products of high-precision positioner fixtures. The SCHUNK company developed a high-precision positioner fixture for large parts, which could provide a clamping force of up to 20 kN and a repeat positioning accuracy of about 5 μm. The System 3R company developed a high-precision positioner fixture for small parts and its highest repeatability positioning accuracy could reach 2 μm [21].

The literature review indicates that much work has been conducted for developing positioner fixtures. However, there are two shortcomings. First, existing positioner fixtures have a low repeat positioning accuracy and it is difficult to meet the requirements of precision machining, especially for the precision machining of precision microparts. Secondly, much research related to fixture design has concentrated on the design method, deformation analysis, stability evaluation and fixture repeatability. There is relatively little research on the error calculation and analysis of high-precision positioner fixtures, especially the allowable machining tolerance values of key parts, which further inhibits development. The structure of this paper is as follows. Firstly, the configuration of the high-precision positioner fixture is presented in Section 2. The calculation and analysis of the positioning error of the positioner fixture and the design of the machining tolerance with important parts are described in Section 3. The repeated positioning accuracy and stiffness test experiments of the proposed positioner fixture are performed in Section 4. Finally, conclusions are drawn in Section 5.

## 2. Design of the High-Precision Positioner Fixture

**Figure 1:**
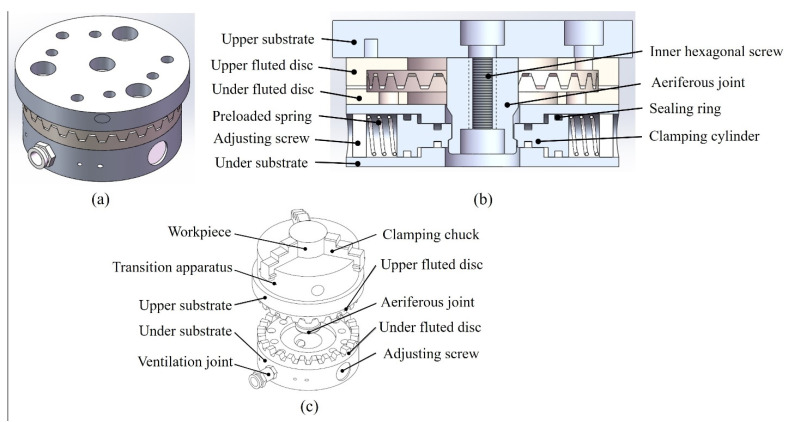
(**a**) Three-dimensional structure diagram of the proposed high-precision positioner fixture. (**b**) Sectional view of the proposed positioner fixture. (**c**) Structure diagram of the proposed positioner fixture with the workpiece.

A three-dimensional illustration of the proposed high-precision positioner fixture is shown in Figure 1. A high-precision end-toothed disc was used to achieve the high repeated positioning accuracy and the high stiffness clamping of the positioner fixture was attained through the matching convex–concave structure, as shown in Figure 1b. In addition, the pneumatic clamping method was selected to achieve the advantages of the positioner fixture of a short positioning time and simple operation. The structure diagram of the proposed positioner fixture with the workpiece is shown in Figure 1c and was divided into upper and lower parts. The lower part was composed of the under substrate, under fluted disc, ventilation joint and adjusting screw. The under fluted disc, ventilation joint and adjusting screw were fixed connected to the under substrate. A clamping cylinder and preloaded spring were also installed inside the under substrate, as shown in Figure 1b. The under substrate was connected to the workbench of the machine tool or testing equipment. The upper part was composed of the upper substrate, upper fluted disc, transition apparatus, aeriferous joint and workpiece. The upper fluted disc, transition apparatus and aeriferous joint were fixed connected to the upper substrate. It should be noted that the workpiece was mounted on the upper substrate through the transition apparatus and the shape of the workpiece was determined by the transition apparatus. An inner hexagonal screw was installed inside the upper substrate and was used to connect the upper substrate and the aeriferous joint, as shown in Figure 1b. The operation steps of the proposed positioner fixture were as follows: in the initial state, the aeriferous joint was not ventilated, the spring pushed the clamping cylinder to the center and the clamping cylinder cooperated with the aeriferous joint to complete the clamping under the action of the spring force. At this time, the upper and under fluted gear discs were in a stable meshing state. When the aeriferous joint was ventilated and the air pressure was greater than the spring force, the clamping cylinder moved from inside to outside. At this time, the positioner fixture was in the unloaded state and the upper fluted gear disc part could be removed, as shown in Figure 1c. It should be noted that the positioner fixture needed to be ventilated during the process of removing and putting in the upper fluted gear disc part. In the proposed positioner fixture, the workpiece was completely positioned. It is worth noting that the adjustment of the clamping force applied by the clamping cylinder to the aeriferous joint could be achieved by regulating the adjusting screw. Thus, the proposed structure of the positioner fixture met the requirements of a high repeated positioning accuracy, high stiffness, short positioning time and simple operation.

## 3. Analysis and Calculation of the Positioning Error

The positioning error of the fixture was numerically equal to the machining error caused by the inaccurate positioning of the workpiece on the fixture. The clamping error between the workpiece and the positioner fixture and the position and size errors between the positioning elements of the positioner fixture were the main factors leading to the positioning error of the positioner fixture [5,22]. In this paper, a high-precision end-toothed disc was used to achieve a high repeated positioning accuracy and the positioning accuracy of the proposed positioner fixture was permitted to be less than 1 μm. It should be noted that the positioner fixture could be equivalent to the indexing operation of the two end-toothed discs when realizing the indexing. Therefore, the error of the end-toothed disc could be calculated according to the error theory of the end-toothed disc. The positioning error of the end-toothed disc mainly included three parts: a cumulative error of the tooth pitch, a tooth alignment error and an error of the tooth profile half-angle [23].

### 3.1. The Cumulative Error of the Tooth Pitch

In the end gear disc, the tooth pitch was the length of the arc between two adjacent teeth on the same side tooth profile with any circumference. The pitch error was the deviation between the actual pitch and the standard pitch. The single pitch error can be written as:

(1) $$Vp_{i}=p_{i}-p$$

where V$p_{i}$ is the tooth pitch error of the *i*-th tooth, $p_{i}$ is the actual pitch of the tooth and p is the standard pitch of the tooth. When the end gear disc crosses *k* teeth, the cumulative error of the tooth pitch (V$p_{\Sigma }$) is expressed by:

(2) $$Vp_{\Sigma }=\Sigma Vp_{i}-kp.$$

**Figure 2:**
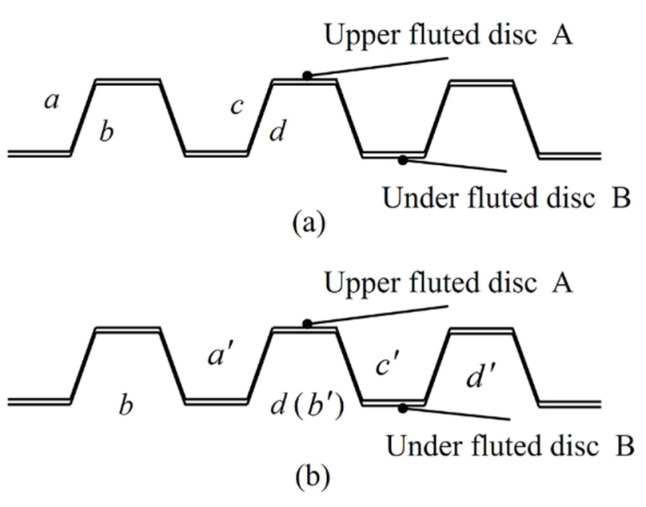
The meshing state of the end gear. (**a**) Before rotating. (**b**) After rotating.

As shown in Figure 2, the under fluted disc was fixed and the upper fluted disc performed a rotating movement. When the upper and under fluted gear discs were engaged, the tooth profiles of the upper and under fluted gear discs were coupled in a one-to-one correspondence.

When the upper fluted gear disc rotates through *k* teeth, the cumulative error value of the tooth pitch of the ab meshing tooth pair ($Vp_{ab}$) can be written as:

(3) $$Vp_{ab}=p_{bb’}-p_{aa’}=Vp_{bb’}-Vp_{aa’}$$

where $Vp_{aa’}$ is the error of the corresponding point and the standard position after the tooth (a) has rotated through *n* teeth. Similarly, the cumulative error value of the tooth pitch of the *cd* meshing tooth pair ($Vp_{cd}$) can be expressed by:

(4) $$Vp_{cd}=p_{dd’}-p_{cc’}=Vp_{dd’}-Vp_{cc’}.$$

If $Vp_{ab}$ is equal to $Vp_{cd}$, then the mating tooth surface *ab* and the mating tooth surface *cd* have not rotated relative to each other during the positioning process. At this time, the angle error $V\varphi_{\alpha }$ is zero. If $Vp_{ab}$ does not equal $Vp_{cd}$, then the mating tooth surface *ab* and the mating tooth surface *cd* are not in contact at the same time. In order to ensure that both tooth surfaces were in contact, the upper and under fluted discs rotated relatively. At this time, the cumulative error of the tooth pitch can be written as:

(5) $$Vp_{\Sigma }=\frac{1}{2}\left(Vp_{ab}+Vp_{cd}\right).$$

The pitch deviation of each tooth is Vp (μm) and the pitch deviation of a single tooth can be regarded as an independent random variable that conforms to the normal distribution and can be expressed as:

(6) $$Vp\sim \left(\mu ,\sigma_{p}{}^{2}\right).$$

Equation (5) can then be written as:

(7) $$Vp_{\sum }\sim \left(0,\frac{1}{4}\left(\sigma_{p_{ab}}{}^{2}+\sigma_{p_{cd}}{}^{2}\right)\right).$$

According to the error statistical analysis method, the actual machining error of the part can be set to conform to the normal distribution. According to this principle, Equation (7) is valid.

(8) $$\pm 3\sqrt{\frac{1}{4}\left(\sigma_{p_{ab}}{}^{2}+\sigma_{p_{cd}}{}^{2}\right)}\leq \pm 0.5\times 10^{-3}.$$

Suppose $\sigma_{p_{ab}}$ is equal to $\sigma_{p_{cd}}$, then the cumulative deviation of the tooth pitch is 1.4 μm and its upper and under deviations are both 0.7 μm.

### 3.2. The Tooth Alignment Error

**Figure 3:**
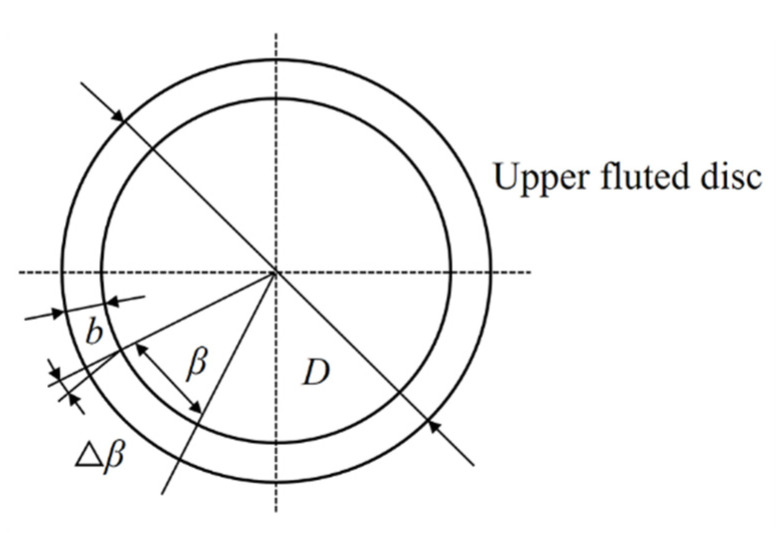
Schematic diagram of the tooth direction error.

The tooth alignment error refers to the angle between the standard tooth direction line and the actual tooth direction line, as shown in Figure 3. In the actual situation, there were tooth alignment errors on the upper and under fluted discs.

In the end gear disc, the tooth pitch was the length of the arc between two adjacent teeth on the same side tooth profile with any circumference. The pitch error was the deviation between the actual pitch and the standard pitch and the single pitch error can be written as:

(9) Vb=btanVβ

where b is the width of a single tooth, β is the circumferential angle corresponding with a single tooth and $\beta =\frac{2\pi }{Z}$. The tooth alignment error ($V\varphi_{\beta }$) can then be written as:

(10) $$V\varphi_{\beta }=\frac{2b\tan V\beta }{D}$$

where D is the outer diameter of the upper fluted disc. When the movement error was within 1 μm (Vb≤1 μm), Equation (9) could be written as:

(11) $$V\beta \leq \arctan\frac{Vb}{b}=1.59\times 10^{-3}.$$

According to the inspection specification for involute cylindrical gears, when the actual tooth profile is tested, the tooth direction deviation is calculated based on the maximum deviation ($VF_{\beta }=V\beta \times b$) between the actual tooth direction line and the standard tooth line in the effective length. It is assumed that the tooth orientation error conforms to a normal distribution so the tooth alignment error can be expressed by:

(12) $$V\beta ∼\left(0,\frac{\sigma_{\beta }^{2}}{b^{2}}\right).$$

(13) $$\pm \frac{3\sigma_{\beta }}{b}\leq \pm 7.95\times 10^{-4}.$$

The tooth alignment error is then 2.12 μm and its upper and under deviations are both 1.05 μm.

### 3.3. The Error of Tooth Profile Half-Angle

The tooth profile angle of the end gear disc refers to the angle between the left and right tooth surfaces of the same tooth. The tooth profile angle directly affects the accuracy of the end gear disc. Usually, the smaller the tooth profile angle, the higher the indexing accuracy and a better positioning stability of the end gear disc. However, when the tooth profile angle is too small, it is easy to increase the axial runout of the gear meshing circle, reduce the stiffness of the tooth root part and increase the amount of deformation, resulting in the decrease in the indexing accuracy of the end gear disc. Therefore, in comprehensive consideration, the tooth profile angle was selected as 60° in this paper. According to the different deviations of the half-angle error of the upper and under fluted discs, it could be divided into the following four types.

**Figure 4:**
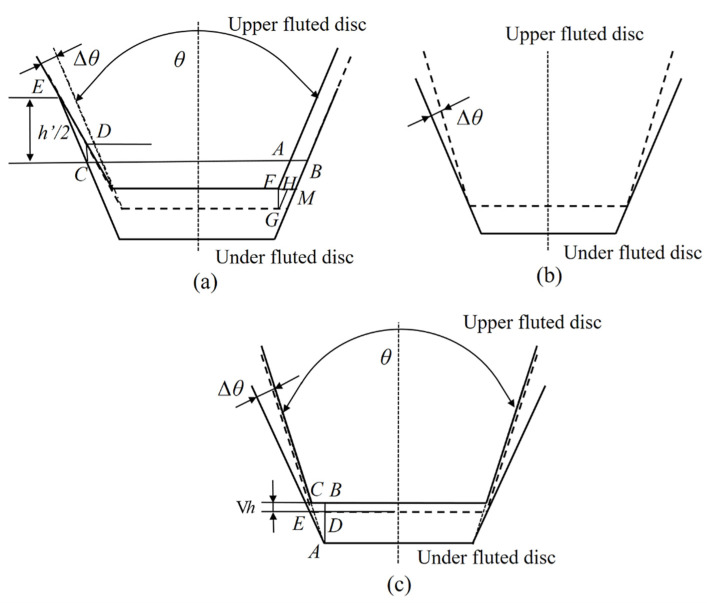
Schematic diagrams of tooth profile half-angle errors in the meshing state. (**a**) The under fluted disc is standard and the deviation of the actual half-angle of the upper fluted disc is biased to the outside of the axis. (**b**) The under fluted disc is standard and the deviation of the actual half-angle of the upper fluted disc is biased to the inside of the axis. (**c**) The upper fluted disc is standard and the deviation of the actual half-angle of the under fluted disc is biased to the outside of the axis.

(a) Type 1: The under fluted disc is standard. The deviation of the actual half-angle of the upper fluted disc from the standard half-angle is Δθ and it is biased to the outside of the axis, as shown in Figure 4a. The center line of the end gear is set as the reference and the distance between the center line and the tooth tip of the under fluted disc is h’/2 where h’ is the working height of the end gear disc and the design size is 4 mm. It can be seen from Figure 4 that the length of the rotation arc around the axis can be expressed as:

(14) $$l_{FH}=l_{AB}/2=l_{CD}\tan\frac{\theta }{2}.$$

The movement distance along the axis is $l_{FG}$ and $l_{FG}=l_{CD}$.Therefore, the corner error caused by the half-angle of the tooth profile can be written as:

(15) $$V\varphi_{\frac{\theta }{2}}=\frac{l_{FH}}{d\pi }\times 360{}^{\circ}=\frac{360{}^{\circ}}{d\pi }l_{CD}\tan\frac{\theta }{2}.$$

As Δθ is extremely small and $\frac{l_{CD}}{\sin\Delta \theta }=\frac{l_{EC}}{\sin\left(180{}^{\circ}-\left(\Delta \theta +\frac{\theta }{2}\right)\right)}$, the corner error caused by the half-angle of the tooth profile can be expressed as:

(16) $$\Delta \varphi_{\frac{\theta }{2}}=\frac{180{}^{\circ}h'\Delta \theta }{\pi d\cos^{2}\frac{\theta }{2}}.$$

When the left and right tooth surfaces of the single tooth have the tooth profile half-angle error, it can be written as:

(17) $$V\varphi_{\frac{\theta }{2}}=\frac{180{}^{\circ}h’}{\pi d\cos^{2}\frac{\theta }{2}}\left(\Delta \theta_{L}\pm \Delta \theta_{R}\right)$$

where $\Delta \theta_{L}$ is the tooth profile half-angle error of the left tooth surface and $\Delta \theta_{R}$ is the tooth profile half-angle error of the left tooth surface. Therefore, the axial displacement error caused by the tooth profile half-angle can be expressed as:

(18) $$l_{FG}=\frac{h’}{2\sin\frac{\theta }{2}\cos\frac{\theta }{2}}\left(\Delta \theta_{L}\pm \Delta \theta_{R}\right).$$

(b) Type 2: The under fluted disc is standard. The deviation of the actual half-angle of the upper fluted disc from the standard half-angle is Δθ and it is biased to the inside of the axis, as shown in Figure 4b. In this case, an error of the tooth profile half-angle does not occur.

(c) Type 3: The upper fluted disc is standard. The deviation of the actual half-angle of the under fluted disc from the standard half-angle is Δθ and it is biased to the inside of the axis. In this case, the calculation method is the same as Type 1 and it can be calculated by changing the corresponding parameters.

(d) Type 4: The upper fluted disc is standard. The deviation of the actual half-angle of the under fluted disc from the standard half-angle is Δθ and it is biased to the outside of the axis, as shown in Figure 4c. In this case, the upper fluted disc moves vertically downwards and $l_{BC}=l_{DE}$. The length of the rotation arc around the axis can then be expressed as:

(19) $$\Delta h=l_{BD}=l_{AB}-l_{AD}=l_{BC}\left(\frac{1}{\tan\frac{\theta }{2}}-\frac{1}{\tan\left(\frac{\theta }{2}+\Delta \theta \right)}\right).$$

Based on the above analysis, it could be seen that the maximum axial movement error caused by the tooth profile half-angle error occurred in Type 1. Usually, the angle deviation could not be obtained directly through the tooth profile half-angle error. According to the inspection specification of the gear tooth profile deviation, it could be obtained by measuring the maximum tooth profile deviation $\sigma_{\theta }$ (μm) of the effective tooth height and the angle error could be expressed as:

(20) $$\Delta \theta =\frac{\sigma_{\theta }}{h^{\prime }}.$$

The length of the rotation arc around the axis could then be written as:

(21) $$l_{FG}\sim \left(0,\frac{\sigma_{\theta_{L}}{}^{2}+\sigma_{\theta_{R}}{}^{2}}{{\left(h^{\prime }\right)}^{2}}\right).$$

(22) $$\pm 3\sqrt{\frac{\sigma_{\theta_{L}}{}^{2}+\sigma_{\theta_{R}}{}^{2}}{{\left(h^{\prime }\right)}^{2}}}\leq \pm 5\times 10^{-4}.$$

Suppose $\sigma_{\theta_{L}}$ is equal to $\sigma_{\theta_{R}}$; the cumulative deviation of the tooth pitch is then 2.82 μm and its upper and under deviations are both 1.41 μm.

From the above analysis, it could be observed that the tooth alignment error and the tooth profile half-angle error had a small effect on the total error and the allowable tolerance ranges were relatively large under the same target accuracy. The cumulative error of the tooth pitch had a great influence on the total error; its allowable error value was small and the error value should be controlled in the machining process.

## 4. Results and Discussion

### 4.1. Experiment to Test the Repeated Positioning Accuracy

**Figure 5:**
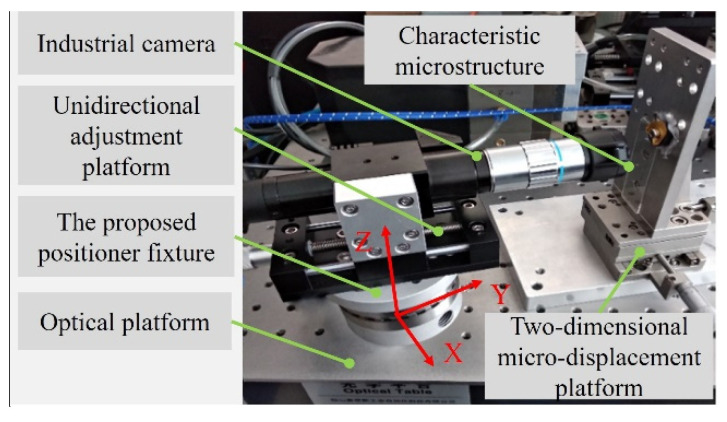
Experimental setup for the repeated positioning accuracy test.

In order to validate the rationality of the configuration of the high-precision positioner fixture and verify the results of the theoretical calculation, a high-precision positioner fixture that met the tolerance requirements was fabricated and a repeated positioning accuracy test of the positioner fixture was performed. Significantly, the test results were affected by the machine tool movement error and tool manufacturing error when the positioning accuracy of the positioner fixture was tested under the cutting state. Therefore, a non-contact test method was used in this experiment. Figure 5 shows the experimental setup of the repeated positioning accuracy test of the proposed positioner fixture. A high-precision industrial camera was fixed on the positioner fixture through a unidirectional adjustment platform and the positioner fixture was fixed on the optical platform. The industrial camera could be moved back and forth through the unidirectional adjustment platform to achieve a precise focusing. A characteristic microstructure sheet was placed on the front end of the industrial camera and it was fixed on the optical platform through a two-dimensional microdisplacement platform. The resolution of the two-dimensional microdisplacement platform in both the x direction and the z direction was 1 μm. The array structure of the characteristic microstructure sheet was 100 μm × 100 μm square grid holes. The machining accuracy of the grid holes was better than 0.1 μm, indicating that the accumulated error caused by the inaccuracies of the grid holes was less than 0.1 μm, which satisfied the requirement of this experiment.

The specific operation method of this experiment was as follows. First, the industrial camera was placed on the proposed positioner fixture and the camera was precisely focused by adjusting the displacement platform. Second, the camera was picked up (including the upper fluted gear disc part) and then put down in its original position. Through this process, the repeated positioning errors occurred and were manifested as changes in the imaging position of the characteristic structure. By processing the image, the displacement error values along the x direction and the z direction could be obtained. The above test experiment was repeated many times to obtain the variation amplitude of the error value and this was the repeated positioning accuracy of the proposed positioner fixture. It should be noted that when the imaging was blurry, the uncertainty error of the image processing was measured by the quantitative movement of the microdisplacement platform. The results demonstrated that the uncertainty error was less than ±0.1 μm, which indicated that the error value along the y direction was small and therefore it was feasible to judge the position in the x and z directions through the image processing method proposed in this paper. The error value along the y direction was shown as the change in the image depth; that is, the resulting image was shown as “clear” and “blurred”. The displacement error value along the y direction could not be obtained by the image processing. The base of the positioner fixture rotated 90° along the z-axis and the direction of the measuring lens remained unchanged. The images were obtained in the same way so that the error along the x direction was the original y direction error.

**Figure 6:**
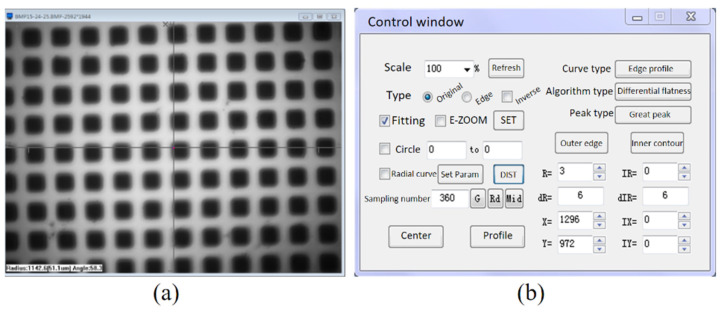
(**a**) The experimental image obtained by the industrial camera. (**b**) The operating interface of the developed image processing software.

After the measurement experiments of the repeated positioning accuracy of the proposed positioner fixture, experimental images were obtained and the image processing was performed. As shown in Figure 6, image processing software was used to dispose of the collected images. The scale parameter was set to 0.045 μm/pixel according to the resolution of the camera. The coordinate position of the cursor in the image was recorded so that the distance and coordinate values of the cursor relative to the origin could be obtained. In order to reduce random errors and improve the accuracy of the test, the repeated positioning accuracy of the positioner fixture was tested 30 times.

**Figure 7:**
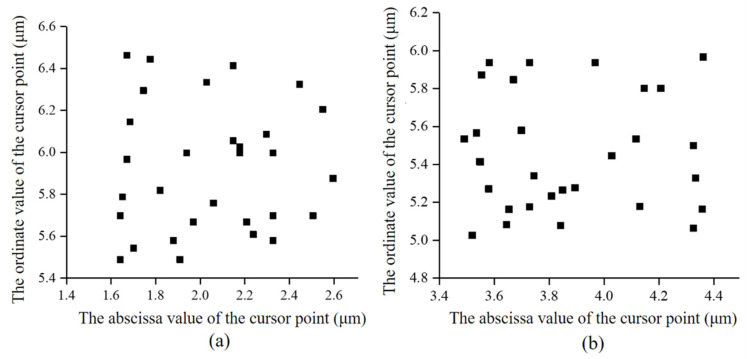
Scatter plot of the cursor points. (**a**) The cursor point distribution before rotation. (**b**) The cursor point distribution after rotating by 90 degrees.

Figure 7a is the cursor point distribution before rotation, which indicated that the repeated positioning accuracy of the positioner fixture proposed in this paper was 0.96 μm in the horizontal (x-axis) direction and 1.0 μm in the vertical (z-axis) direction. The base of the positioner fixture rotated 90° along the z-axis and the direction of the measuring lens remained unchanged. The new distribution diagram of the cursor points was then obtained, as shown in Figure 7b. It could be ascertained that the repeated positioning error of the proposed positioner fixture along the horizontal (y-axis) direction was 0.9 μm and this error along the vertical (z-axis) direction was 0.96 μm. It should be noted that there were two reasons causing the slight deviation of the two test results of the repeated positioning error along the vertical (z-axis) direction. First, there was a slight movement between the camera and the connector when the camera (including the upper fluted gear disc part) was repeatedly picked up and put down. Second, there was a slight deviation in the selection of the cursor point under the image processing process. In summary, the repeated positioning accuracy of the proposed positioner fixture in the x, y and z directions were ±0.48 μm, ±0.45 μm and ±0.49 μm, respectively. The test results indicated that the proposed positioner fixture had a high repeated positioning accuracy and its value was less than 1 μm, which met the initial design requirements.

### 4.2. Stiffness Measurement of the Proposed Positioner Fixture

**Figure 8:**
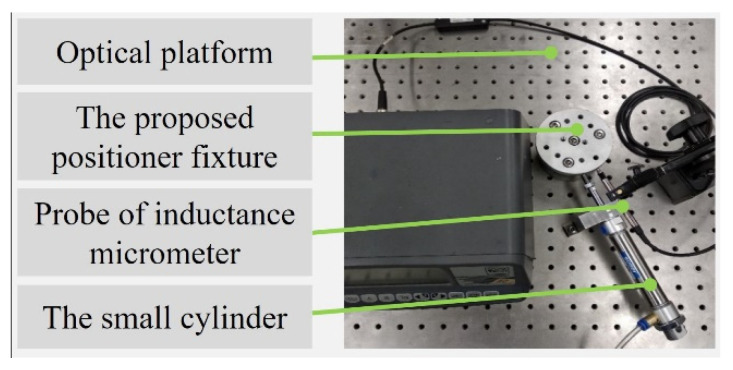
Experimental setup for the stiffness measurement.

In order to verify that the proposed positioner fixture could maintain a high positioning accuracy under working conditions, it was necessary to conduct a stiffness test on the proposed positioner fixture. The experimental setup of the stiffness measurement is shown in Figure 8. The testing devices mainly included the proposed positioner fixture, an inductance micrometer, a small cylinder and an optical platform. The cylinder was used as a force application device and the applied force value could be continuously changed from 0 to 100 N by precisely adjusting the value of the air pressure. The inductance micrometer selected in this experiment was the TT80 inductance micrometer, which is produced by the Swiss TESA company. The resolution of the inductance micrometer was 0.1 μm, which met the needs of the experiment.

**Figure 9:**
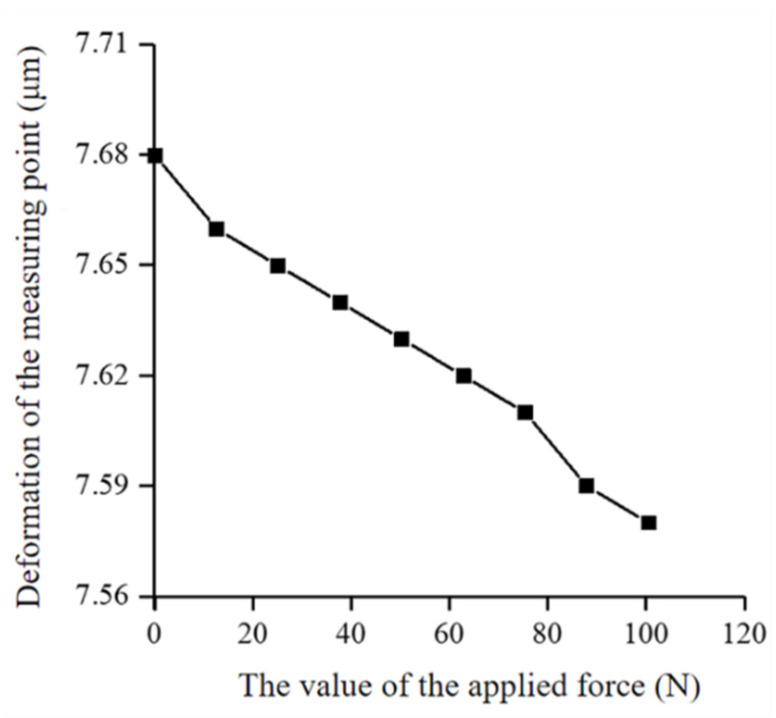
The deformation of the measured point varies with the applied force.

Figure 9 is the numerical curve diagram of the different values of the applied force and displacement of the measured point. The equation of this curve could be written as y=−0.0009x+7.676 and the slope of the curve was −0.0009. According to the relationship between the applied force and the displacement, the stiffness of the proposed positioner fixture could be calculated to be 1050.5 N/μm, which was larger than the previous positioner fixture of the same type. It is worth noting that the greater the stiffness of the proposed positioner fixture, the smaller the deformation generated during the machining process, which is beneficial for precision machining. When the measuring head and the direction of the applied force were changed in the horizontal plane, the stiffness value of the positioner fixture was almost unchanged. When the measuring head and the direction of the applied force were changed along the z direction, the test results showed that the stiffness of the positioner fixture in the vertical direction was much greater than in the horizontal direction. The test results showed that the proposed positioner fixture had a higher stiffness. In summary, the positioner fixture proposed in this paper met the requirements of a high repeated positioning accuracy and high stiffness.

## 5. Conclusions

Based on the configuration design analysis, the theoretical calculations and the performance verification experiments of the proposed positioner fixture, the following conclusions can be drawn:

1. An innovative high-precision positioner fixture was proposed and investigated in this paper. The proposed positioner fixture had the advantages of a short positioning time and simple operation. The high-precision end-toothed disc was the key point for achieving the high repeated positioning accuracy. The mathematical models of the cumulative error of the tooth pitch, the tooth alignment error and the error of the tooth profile half-angle of the end-toothed were analyzed. The analysis results indicated that the tooth alignment error and the tooth profile half-angle error had a smaller effect on the total error whereas the cumulative error of the tooth pitch had a greater influence. The allowable tolerance values of the cumulative error of the tooth pitch, the tooth alignment error and the error of the tooth profile half-angle of the end-tooth were given.
2. The repeated positioning accuracy test results showed that the proposed positioner fixture had a high repeated positioning accuracy and its repeated positioning accuracy in the x, y and z directions were ±0.48 μm, ±0.45 μm and ±0.49 μm, respectively, which was significantly higher than previous positioner fixtures. The stiffness test results showed that the stiffness of the proposed positioner fixture was 1050.5 N/μm, which was large enough for a fixture usually used in the field of precision machining. The above analysis results indicate that the designed positioner fixture had the advantages of a high repeated positioning accuracy, high stiffness, short positioning time and simple operation.
